# The Role of Myocardial Revascularization in Ischemic Heart Failure in the Era of Modern Optimal Medical Therapy

**DOI:** 10.3390/medicina61081451

**Published:** 2025-08-12

**Authors:** Ioana-Paula Blaj-Tunduc, Ciprian Marcel Ioan Brisc, Cristina Mihaela Brisc, Dana-Carmen Zaha, Cristiana-Magdalena Buştea, Vlad-Victor Babeş, Teodora Sirca-Tirla, Francesca-Andreea Muste, Elena-Emilia Babeş

**Affiliations:** 1 Doctoral School of Biomedical Sciences, Faculty of Medicine and Pharmacy, University of Oradea, 410087 Oradea, Romania; blaj.ioanapaula@student.uoradea.ro (I.-P.B.-T.); briscciprian@uoradea.ro (C.M.I.B.); brisccristina@yahoo.com (C.M.B.); dzaha@uoradea.ro (D.-C.Z.); sirca.teodorabianca@student.uoradea.ro (T.S.-T.); furko.francescaandreea@student.uoradea.ro (F.-A.M.); eebabes@uoradea.ro (E.-E.B.); 2 Department of Medical Disciplines, Faculty of Medicine and Pharmacy, University of Oradea, 410073 Oradea, Romania; 3 Department of Preclinical Disciplines, Faculty of Medicine and Pharmacy, University of Oradea, 410073 Oradea, Romania

**Keywords:** ischemic heart failure, chronic coronary syndrome, complete revascularization, depressed ejection fraction, percutaneous coronary intervention

## Abstract

*Background/Objectives*: Heart failure (HF) with reduced ejection fraction (EF) has, in more than 50% of cases, an ischemic etiology and continues to be associated with increased mortality and morbidity despite all the progress registered in the field of medical therapy and interventional revascularization. Myocardial revascularization is extensively used in clinical practice based on the traditional concept that it can improve myocardial function and outcome in ischemic HF. This review is aimed at presenting current knowledge regarding revascularization in patients with chronic ischemic HF and reduced EF. *Methods*: The impact of revascularization on symptomatology, left ventricle reverse remodeling, major adverse cardiac events (MACEs), and the role of complete revascularization and of percutaneous interventional revascularization in chronic total occlusion (PCI-CTO) were analyzed. The best therapeutic strategies, revascularization and/or optimal medical therapy (OMT), are debated in different categories of patients, in order to identify who will benefit more from revascularization strategies. *Results*: Based on the long-term results of the STICH trial incorporated in the guidelines with a class I-b recommendation, coronary artery bypass graft (CABG) remains the main modality of revascularization for prognostic improvement in ischemic HF with multivessel disease. But real-life patients are usually old with multiple comorbidities and high surgical risk. In this category, the Heart Team opinion is required to evaluate the probability of complete revascularization and to choose between percutaneous coronary intervention (PCI) and CABG according to clinical status and coronary anatomy. *Conclusions*: However, until further studies are available, the results of the REVIVED-BCIS2 trial encourage OMT over PCI in patients with ischemic cardiomyopathy. The available randomized controlled trials (RCTs) showed improved angina and quality of life in PCI-CTO versus OMT, but the effect on MACEs was not demonstrated.

## 1. Introduction

Heart failure (HF) is defined as a syndrome with multiple etiologies. The most common etiology is represented by coronary artery disease (CAD), which provides a major contribution to morbidity rate, hospitalizations, and mortality despite scientific discoveries over the last decade and preventive measures [1,2]. Myocardial damage due to multivessel disease (MVD) leads to impaired left ventricular (LV) systolic function, adverse remodeling, reduced ejection fraction (EF), causing ischemic HF, and higher mortality rates compared to non-ischemic HF [2].

Over the last three decades, ischemic heart disease has accounted for approximately 197 million cases and over 9 million deaths globally, according to Global Burden of Disease data [3]. Although mortality has declined in some regions, for instance, from 14.6% in 2008 to 7.8% in 2022 in Italy [4], the prognosis remains poor in patients with EF < 50%, as shown in long-term registries such as the Framingham Heart Study and the Swedish Heart Failure Registry [5,6]. Since trials usually enroll younger and healthier patients, we assume that outcomes are often worse in real-world settings than those reported in clinical trials [7,8].

Myocardial ischemia affects both systolic and diastolic function due to recurrent episodes of oxygen supply–demand imbalance, leading to adverse LV remodeling and poor outcomes for patients with ischemic HF [9]. Revascularization encompasses coronary artery bypass grafting (CABG) or percutaneous coronary intervention (PCI) and may reverse these effects, improve EF and quality of life, and reduce hospitalization rates [9,10]. However, the benefits of myocardial revascularization and the role of ischemia and viability assessment remain controversial.

Current guidelines recommend complete revascularization (CR) without specific recommendations in patients with chronic total occlusion (CTO) [1]. A Surgical Treatment for Ischemic Heart Failure—STICH follow-up trial revealed that CABG in addition to optimal medical therapy (OMT) for ischemic cardiomyopathy did not confer a better outcome compared to medical treatment alone 2 years after the procedure. However, many patients are unsuitable for CABG due to patient-related factors (age, comorbidities), complex anatomy, or prior interventions [11,12,13]. In the meantime, due to important advances in the field of interventional revascularization, extensive revascularization with PCI is appealing and promises to be a better alternative for these high-risk patients [12]. PCI is a less invasive option with favorable procedural outcomes, but its role in patients with HF and reduced EF remains unclear because of underrepresentation in randomized trials [14].

The randomized controlled trials (RCTs) dedicated to patients with chronic coronary syndrome (CCS) have excluded, in general, patients with ischemic HF, so it is unclear if their findings apply to this category of patients [15,16,17]. Notably, the REVIVED-BCIS2 trial did not prove any additional benefit over OMT in patients with extensive CAD and severely impaired LV systolic function [18].

Achieving CR may improve prognosis, but it is frequently limited by anatomical and clinical factors [19]. Observational data suggest that incomplete revascularization (ICR) is associated with higher mortality in HF patients [20]. The advantage of CAGB in MVD is linked to the higher likelihood of achieving CR.

CTOs are present in about 25% of CAD patients, and, historically, these lesions were left untreated because there is a lack of knowledge regarding their prognostic significance, especially in patients with HF [21]. However, recent advancements in interventional cardiology have made successful PCI of CTOs possible in 90% of cases with low complication rates [22]. Still, these patients often carry a high procedural risk and poorer outcomes, especially when CTOs coexist with ST-elevation myocardial infarction (STEMI) or when the occlusion compromises perfusion of the infarct-related artery [22,23,24]. Data from HORIZONS-AMI and other large trials have demonstrated worse outcomes in patients with concurrent CTOs [25,26,27], particularly in diabetics [28].

While CTO PCI improves symptoms and quality of life, its impact on major adverse cardiac events (MACEs) such as death, myocardial infarction (MI), survival rate, and repeat revascularization remains unclear in RCTs [29,30,31,32,33].

This review aims to present current knowledge regarding revascularization in patients with chronic ischemic HF and reduced LVEF. It will explore the impact of revascularization via CABG or PCI, with particular attention to the role of CTO interventions and the influence of CR versus ICR on clinical outcomes. The goal is to identify a specific patient profile that may have the greatest benefit from revascularization. Furthermore, this review will examine existing data comparing revascularization in addition to OMT versus OMT alone, with the purpose of evaluating the overall clinical outcomes and the supplementary benefit that is derived from revascularization strategies.

## 2. Materials and Methods

This review was conducted as a narrative synthesis of the selected literature and scientific publications addressing revascularization strategies, CTO, and OMT in the management of ischemic HF. The purpose was to explore the impact of these interventions on prognosis, mortality, morbidity, LV remodeling, complications, and hospitalization rates. There were no limitations regarding publication date; both recent and old studies were included to provide a comprehensive historical and clinical perspective.

The databases used for selecting relevant articles were E-nformation, PubMed, Cochrane Library, and Web of Science. A combination of relevant keywords (e.g., “ischemic heart failure”, “chronic coronary syndrome”, “complete revascularization”, “depressed ejection fraction”, “percutaneous coronary intervention”), along with Medical Subject Headings (MeSH) terms, was utilized for the retrieval of pertinent studies. The search strategy and article selection process followed a methodology resembling PRISMA (Figure S1) (Preferred Reporting Items for Systematic Reviews and Meta-Analyses) style to ensure rigor in study identification, screening, and inclusion. From an initial pool of 895 records, we excluded irrelevant articles and screened 485 articles. A total of 182 were relevant and included in this narrative review. The inclusion criteria encompassed the literature and studies involving ischemic HF and reduced EF, revascularization strategies (PCI, CABG, CTO PCI) or OMT, randomized controlled trials (RCTs), high-quality observational studies, and meta-analyses published in the English language. The exclusion criteria included non-English language articles, studies, and case reports without relevant clinical outcome data or full-text availability. The eligible studies were selected first from the title and abstract, and then a critical analysis of the full-text content was performed in order to extract the most relevant data. To minimize these limitations, a balanced analysis of RCTs, meta-analysis, and selected high-quality observational studies was performed. However, due to study heterogeneity and the evolving management of HF, the results should be interpreted in the context of study limitations.

## 3. Pathophysiology of Ischemic Heart Failure and Chronic Total Occlusion

**Figure 1:**
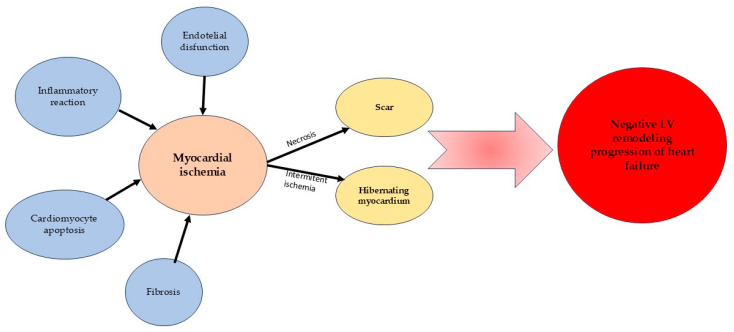
Pathophysiology of ischemic heart failure. Legend: LV—left ventricle.

Half of the cases of HF with reduced EF are with ischemic etiology [7]. Myocardial ischemia is caused by one or more obstructive atherosclerotic plaques, which impair or decrease coronary blood flow [34]. Repeated ischemic events affect the myocardium, producing an inflammatory reaction, important loss of cardiomyocytes, and development of stunning, hibernating myocardium, or cardiomyocyte apoptosis leading to necrosis and, in the final stage, fibrosis [35]. The result of these changes is adverse LV remodeling, dilatation, and impaired LVEF (depicted in Figure 1). All the complications that arise from LV remodeling (decreased EF, rhythm disturbance, mitral ischemic regurgitation) are contributors to worse prognosis, repeated hospitalization, and increased mortality rates in patients with ischemic HF [36]. According to the European Society of Cardiology (ESC) guidelines for the diagnosis and treatment of acute and chronic HF, this disease is classified as follows: HF with reduced EF (less than 40%), mildly reduced EF (41–49%), and preserved EF (≥50%) [37]. Patients with ischemic HF with reduced and mildly reduced EF have an approximately 30% decreased 5-year survival compared with nonischemic HF with reduced EF [38].

CTO can aggravate the progression of HF in patients with ischemic cardiomyopathy and is associated with poor long-term outcomes. The mortality and morbidity remain high in ischemic HF, challenging the practitioner to rethink the management of ischemic HF and search for a new approach to these patients [39]. The definition of CTO is a totally obstructed lumen of one or more coronary arteries for more than 3 months, which can coexist with other coronary artery stenosis [40]. The etiology of CTO is usually an acute thrombotic event, misdiagnosed or untreated [22]. For those who have not experienced a previous MI, the blockage is a result of gradual narrowing of the coronary artery’s lumen over a long period with collateral development, which will partially compensate for the reduced blood flow but is insufficient to sustain myocardial function under stressful circumstances [41]. The myocardial area vascularized by CTO is proarrhythmogenic due to fibrosis, residual ischemia, and hibernating myocardium, even if the collateral circulation is already developed [42]. Patients with ischemic cardiomyopathy and CTO with implanted cardioverter defibrillators have a significantly higher incidence of delivered shocks versus patients without CTO [43].

## 4. Evidence-Based Approaches to Revascularization Strategies in Ischemic Heart Failure with Reduced Ejection Fraction

### 4.1. Patient Selection for Coronary Artery Bypass Grafting Versus Percutaneous Coronary Intervention

#### 4.1.1. Coronary Artery Bypass Graft Surgery in Ischemic Heart Failure with Reduced Ejection Fraction

From the 80s until today, CABG has been the first choice for patients with advanced CAD with impaired LV systolic function and who are eligible for surgical revascularization [44].

The old Coronary Artery Surgery Study (CASS) registry, which was created more than 30 years ago, before the modern era of OMT, enrolled a total of 8221 patients with severe LV dysfunction and CAD. At long-term follow-up (5 years), the survival appeared to be significantly better with CABG compared to medical therapy (68% vs. 54%, *p* = 0.0007), especially in patients with EF ≤ 25%, who had the greatest benefit (63% vs. 54%). In conclusion, although the study had a large sample size, medical therapy was suboptimal compared with current modern therapy, the viability assessment was not performed systematically, and primary endpoints focused on mortality, limiting its clinical applicability to the HF population nowadays [45].

STICH was an RCT from a more contemporary period that enrolled 2112 patients with ischemic HF in New York Heart Association (NYHA) class III-IV, a mean EF of 28%, and a suitable anatomy for CABG without left main disease [14].

In total, 1212 patients were enrolled in hypothesis 1 comparing CABG in addition to medical therapy versus OMT alone. In patients with ischemic HF who underwent CABG, mortality was higher at 30 days but significantly improved in the long term compared with the medical treatment group. Based on trial findings, age significantly influenced CABG benefits, which were diminished in older patients due to several comorbidities. At the per-protocol analysis, all-cause death had a 24% lower rate in the CABG group (*p* = 0.005) [14]. The magnitude of CAD and LV systolic function impairment was correlated with the benefits of surgical revascularization. In STICH, only patients with three vessel disease on OMT had a survival benefit after CAGB compared to OMT alone (HR, 0.79 [95% CI, 0.63–0.99]; *p* = 0.046), while those with less extensive CAD on OMT had no significant survival benefit after CABG (HR, 0.98, [95% CI, 0.73–1.32]; *p* = 0.906) [46]. In addition, a subgroup of patients presented with minimal angina, raising questions about the role of CABG in patients with mild symptoms. Some reported crossovers between treatment arms could have influenced the results; 15% of patients initially assigned to the medical treatment group ultimately performed CABG, and some patients from the CABG group did not undergo the surgical procedure. These crossovers influenced the treatment effect and the interpretation of data.

The first 5 years of follow-up did not show a significant survival benefit for CABG versus OMT (*p* = 0.12), sparking criticism about the insufficient statistical power of the study and leading to a null hypothesis. However, the longer follow-up STICH Extended Study (9.8 years) (STICHES) revealed a 16% reduction in death from any cause in the group with CABG compared to the OMT group (*p* = 0.02) [47]. These findings demonstrate that CABG may offer long-term outcome benefits that are often missed on short-term follow-up intervals. Furthermore, subgroup analyses revealed that three-vessel disease and severely remodeled LV patients (LVEF < 27%, LV end-systolic volume > 78 mL/m^2^) had the greatest benefits after myocardial revascularization [46,47,48]. Mitral regurgitation (MR) was assessed in both groups, and, in the medical therapy group, a high grade of MR was associated with increased mortality. In patients with moderate-severe MR, CABG itself had no significant prognostic impact, but concomitant mitral valve repair was associated with a decreased rate of death versus OMT (HR 0.68) and improved long-term survival [49]. The STICH remains a landmark trial that provided important data regarding the role of surgical revascularization in patients with ischemic cardiomyopathy. But the applicability in contemporary clinical practice is limited by several factors. Firstly, the trial was conducted before the implementation of current optimal guideline-directed medical therapies for HF with reduced EF, especially angiotensin receptor–neprilysin inhibitors (ARNIs) and sodium-glucose co-transporter 2 inhibitors (SGLT2Is). Secondly, the benefits of CABG were not evaluated against PCI, leaving unresolved questions about the potential benefits of less invasive revascularization methods.

Although the STICH trial proved a long-term survival benefit with CABG, no significant improvement in LVEF was observed. In contrast, a retrospective study conducted on 166 patients with ischemic HF and impaired systolic function who underwent CABG revealed that cardiac function and EF were improved in more than half of the patients concomitantly with an increased survival rate [50]. This suggests that surgical revascularization may have a significant contribution to functional recovery, particularly in these patients, but the results should be interpreted with caution, given the study’s limited sample size and lack of a control group.

Evidence from registries and randomized trials until today supports that CABG is superior regarding long-term survival compared to PCI and OMT in high-risk patients with ischemic HF, reduced EF, and extensive CAD. The application of these findings to clinical practice is restricted by several limitations of the current data. As mentioned above, the CASS registry, while influential, was conducted before the adoption of contemporary OMT and did not assess myocardial viability, thus reducing its relevance nowadays. Similarly, the STICH trial had a lot of crossovers and did not incorporate PCI as a comparator. Moreover, the survival benefit of CABG in STICH reached statistical significance only after a follow-up of nearly 10 years, emphasizing the importance of long-term observation to evaluate therapeutic advantage. In conclusion, these findings suggest that further RCTs are necessary to optimize and personalize revascularization strategies in ischemic HF.

#### 4.1.2. Interventional Revascularization in Ischemic Heart Failure with Reduced Ejection Fraction—Revascularization for Chronic Total Occlusion

Interventional revascularization via PCI plays an important role in the management of patients with multivessel CAD. While PCI may provide symptomatic relief and a faster recovery, its prognosis in the long term compared with CABG or OMT is still under debate, especially in patients with MVD or severely reduced EF. Several studies suggest that the extent of coronary revascularization achieved may have an important effect on the outcome. In this context, we will approach the prognosis of CR versus ICR in patients with HF and reduced EF in the section below.

CR may be classified as anatomic, referring to the revascularization of all “significant” coronary lesions defined, in general, as stenosis ≥ 50% or 70%, in vessels with a diameter of at least 2.0 mm, or functional when targeting all the lesions that induce ischemia through physiological assessments such as a fractional flow reserve (FFR) [51]. CR does not include only culprit lesions responsible for acute coronary syndromes (ACSs) but also non-culprit lesions that maintain the residual ischemia [52].

Various data sources from observational studies and clinical trials suggest that CR is usually associated with fewer cardiovascular adverse events [51,53]. A meta-analysis of 89,883 patients with MVD concluded that complete anatomic revascularization was superior to IR with an important reduction in mortality and MI [54].

The SYNTAX Extended Survival study compared outcomes after 10 years from CABG in the CR group, CABG IR group, PCI CR group, and PCI IR group from the patients originally enrolled in the SYNTAX trial [55]. IR was more prevalent in the PCI group compared with the CAGB group (56.6% versus 36.8%) and was correlated with a higher 10-year mortality versus the CABG CR group (33.5% versus 23.7%) [55]. IR is a condition strongly associated with poor long-term outcomes (all-cause death: 50% at a 10-year follow-up versus 22% in patients performing CR; *p* < 0.001) [55]. Among patients with multivessel CAD, particularly in those with complex anatomy of coronary arteries (e.g., a SYNTAX score > 23) [56], CABG was superior to PCI, achieving higher rates of CR. These findings emphasize that the extent and complexity of CAD are important factors in patient selection for the optimal revascularization strategy.

In the context of CTO, a retrospective observational study involving 359 patients further emphasized the prognostic significance of CR. After a median follow-up of 42 months, a lower incidence of MACE was found in patients with CR-CTO, driven by a lower risk of overall mortality. IR-CTO patients had worse outcomes, facing higher rates of HF hospitalizations and MI, highlighting the importance of achieving CR in this category of patients [57].

The risk of mortality and MACEs is significantly lower in CR regardless of whether an anatomical or functional definition of IR is utilized, and the magnitude of risk is correlated with the extent of IR [58,59,60]. A systematic review, which included observational data from large registries with 156,240 patients enrolled, highlighted the prognosis of CR vs. IR in MVD, concluding that CR was associated with a lower risk of death and MACEs [58]. However, certain patient-specific factors may influence the benefit derived from CR. A prospective study on 381 patients with STEMI and MVD assessed the impact of IR on long-term outcomes in patients over 75 years versus those under 75 years [61]. The authors concluded that CR may have significant benefits in younger patients, while, in elderly people, comorbidities and frailty outweigh the benefits of extensive revascularization [61]. A sub-analysis of the PRAISE registry, on more than 20,000 patients with STEMI or non-ST-elevation myocardial infarction (NSTEMI), demonstrated that NSTEMI patients had a more complex clinical picture, more frequent MVD, and prior revascularization interventions but a slightly better preserved LVEF. The 1-year outcomes after discharge were similar between STEMI and NSTEMI after adjustment for clinical factors. However, subgroup analysis suggested that CR may carry greater prognostic significance in NSTEMI, likely due to the more diffuse nature of CAD in this population [62].

Additionally, a recent observational study evaluated the benefit of revascularization in patients with reduced LVEF and intermediate coronary stenoses (stenoses > 40%). The study enrolled 840 patients (206 in the fractional flow reserve (FFR) group and 634 in the revascularization group). The rate of MACEs was higher in the revascularization group (67.5% versus 59.7%) compared to the group where revascularization was deferred based on FFR. Median follow-up was 7 years, and death from all causes was lower in the FFR group (45.6% compared to the revascularization group’s 57.8%, HR-0.65, [95%CL, 0.49–0.85]; *p* < 0.01) [63]. These findings underscore the importance of FFR and raise the question about the potential overtreatment in anatomical-based revascularization strategies, though the non-randomized design of this study restricts definitive conclusions.

In summary, CR continues to be an important therapeutic objective in patients with ischemic heart failure and MVD, correlating with decreased mortality, MACE, and HF-related hospitalizations. Nonetheless, patient-specific factors such as age, comorbidities, frailty, CAD complexity, and physiological lesion significance must be carefully considered in deciding revascularization strategies.

##### Revascularization for Chronic Total Occlusion (CTO): Current Evidence and Clinical Implications

PCI-CTO has been increasingly performed in recent years. Approaching the CTO can be difficult even for the experienced interventional physicians because there is an increased risk of re-occlusion and procedural complications as coronary artery dissection and distal embolization [64]. Historically, CTOs were treated conservatively with antiplatelets, statins, and therapy for improving angina episodes [65]. But nowadays, the introduction of the hybrid PCI technique, first described by Brilakis et al., has markedly improved procedural success by integrating both antegrade and retrograde approaches, allowing for a personalized strategy based on lesion characteristics [66]. There are several anatomical factors that can influence the procedural success and complication rates in CTO interventions. These include the following: proximal cap morphology, lesion length and composition, course and tortuosity of the occluded segment, distal cap characteristics, and the presence and quality of collateral circulation [67,68].

The J-CTO (Japanese Chronic Total Occlusion) score is a standardized technique developed to predict the procedural complexity of PCI-CTO. Based on five parameters—occlusion length (>20 mm), presence of calcification, vessel tortuosity, morphology of the proximal cap (blunt stump), and prior failed attempts—the score estimates the probability of successfully crossing a CTO lesion with a guidewire within 30 min [69]. A low J-CTO score (0–2) is associated with a high success rate of revascularization and a good prognosis [70]. An elevated J-CTO (3–5) score estimates a low success rate, increased technical difficulty, longer procedural time, and a higher likelihood of requiring intravascular ultrasound or rotational atherectomy to upgrade the technique for a better outcome [71]. The predictive utility of the J-CTO score was validated in a multicenter registry involving 650 patients (657 lesions), demonstrating that procedural time increased by around 20 min for each supplementary point in the J-CTO score [71]. Since its introduction, the J-CTO score has been widely used in clinical practice and various studies as a reliable tool for procedural planning and risk stratification in CTO PCI [72].

**Figure 2:**
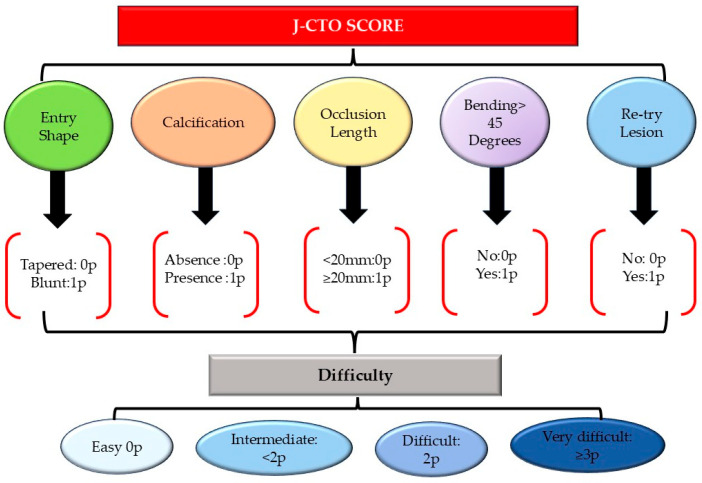
J-CTO score (Japanese Chronic Total Occlusion score).

Figure 2 highlights the components and the computation of the J-CTO score, guiding clinical assessment and procedural planning [73].

Over time, the PCI-CTO technique’s success rate increased, thus reducing the rate of complications, and several centers report a success rate over 80% for CTO lesions, even if the patients who usually perform PCI-CTO have already failed with OMT and have a lot of comorbidities and a large ischemic territory [66,74].

Despite technical innovation, RCTs investigating CTO PCI have produced conflicting results, with most failing to demonstrate survival or MACE benefit over OMT alone.

The EXPLORE-CTO (Evaluating Xience and Left Ventricular Function in PCI on Occlusions After STEMI) was the first randomized trial that compared PCI-CTO, early after primary PCI for STEMI, with medical therapy. At a median follow-up of 10 years, no benefit was demonstrated in terms of MACEs, which occurred in a similar percentage, 25% in the group with CTO PCI, and 24% in the group with no CTO PCI ([HR], 1.11 [95% CI, 0.70–1.76) [75]. However, a subgroup analysis indicated a significant improvement in LVEF in LAD-CTO patients after 4 months versus the group with no CTO PCI (47.2 ± 12.3% vs. 40.4 ± 11.9%, *p* = 0.02), suggesting that lesion location may influence outcomes [31,75,76,77,78,79]. In addition, an increase in systolic thickening in dysfunctional segments after PCI-CTO was demonstrated, especially in patients with well-developed collateral vessels [75,80]. Although further research is needed to draw any firm conclusions, prognostic improvement is expected in patients at increased risk with PCI-CTO on LAD.

The Randomized Multicenter Trial to Compare Revascularization With Optimal Medical Therapy for the Treatment of Chronic Total Occlusions, EUROCTO, demonstrated significant symptomatic relief with CTO PCI but no difference in cardiovascular mortality or MI over a 3-year follow-up [81].

The Impact on Inducible Myocardial Ischemia of PercutAneous Coronary InTervention versus Optimal Medical TheRapy in Patients with Right Coronary Artery Chronic Total Occlusion, the IMPACTOR-CTO trial, reported improvements in symptoms and quality of life but failed to show prognostic benefit [33]. IMPACTOR-CTO was a small RCT that randomized patients with a single occluded coronary artery (RCA), restricting its applicability in more complex MVD CTO [33].

Another open-label multicentric trial, DECISION-CTO (Optimal Medical Therapy With or Without Stenting for Coronary Chronic Total Occlusion), was the largest RCT that randomized 834 patients and compared PCI or no PCI for the qualifying CTO lesion with the option to perform an PCI of obstructive non-CTO lesions at the discretion of the operator. After a follow-up of 4 years, MACEs (death, MI, stroke) were registered in similar proportions in both strategy groups [82,83].

However, all these RCTs had high crossover rates and enrollment challenges, with potentially resulting selection bias. Furthermore, high-risk, severely ill patients with multiple comorbidities were suboptimally represented, compromising the generalizability of these results.

Regional Left Ventricular Function after Stent Implantation in Chronic Total Occlusion—the REVASC trial was an RCT that assessed LV function following PCI-CTO compared to medical therapy alone and failed to prove a considerable improvement of LVEF in PCI patients on a median follow-up period of 6 months. MACEs were reduced at 12 months in the PCI-CTO group compared to the group that did not perform PCI. However, the patients with LVEF < 25% were excluded, and only stable patients were studied [84]. The short follow-up, the small sample size, and exclusion of high-risk patients limit its clinical applicability regarding long-term potential benefit, particularly in patients who may benefit more from revascularization.

In contrast to RCTs, several observational studies and registry analyses have shown improved survival and reduced MACEs following successful CTO PCI, particularly in the following categories: patients with LAD-CTO [85,86], those with significant angina or angina equivalents [87], and in diabetic populations, where OMT alone was linked to higher mortality and revascularization rates [88].

A large-scale registry database, the National Cardiovascular Data Registry CathPCI Registry linked to Medicare (>550,000 patients), demonstrated lower mortality, hospital readmissions, and repeat revascularization in CTO PCI patients compared to high-risk non-CTO PCI cases [89].

A positive impact of PCI-CTO on LV remodeling and systolic function was suggested in a meta-analysis of 34 observational studies involving 2375 patients. The analysis demonstrated a significant improvement in LVEF and a notable reduction in LV end-systolic volume over a mean follow-up period of 7.9 months. Extended follow-up studies are required since the recovery of hibernating myocardium starts around 3–6 months after revascularization, and the systolic function recovery can continue up to 24 months, as was demonstrated in CMR studies [90,91]. The short follow-up duration, absence of a randomized control, and reliance on an observational study limit the applicability to clinical practice.

A recent meta-analysis included 16 studies with 11,314 patients and was aimed at evaluating the outcome of patients who performed CTO-PCI versus patients with OMT alone [29]. In observational studies, CTO-PCI yielded a lower all-cause and cardiovascular mortality (OR: 0.45 and 0.58) versus OMT alone [29]. RCTs showed no significant improvement in MACE (OR:0.71), recurrent MI (OR:0.71), recurrent PCI (OR: 0.61), or stroke (OR: 0.61) [29]. However, a reduction in MACEs, recurrent MI, and stroke was found, although it was not statistically significant [39].

The ongoing trial, an international randomized trial on the effect of revascularization or optimal medical therapy of chronic total coronary occlusion with myocardial ischemia-the ISCHEMIA trial, aims to demonstrate if CTO-PCI, in addition to OMT, improves MACEs and quality of life and decreases hospitalization for HF compared to OMT alone in patients with extensive ischemia. Patients will be classified based on ischemia burden and symptom severity, implementing a run-in phase with OMT and non-CTO lesion revascularization before randomization. Different from previous trials, ISCHEMIA-CTO promotes assessment of ischemia and specific and stratified endpoints such as MACE, arrhythmias, HF hospitalization, and postponed randomization, correcting many flaws from other studies. Its results may clarify the role of CTO PCI, revealing the true prognostic impact [92].

In conclusion, although observational studies reported a decreased mortality in PCI-CTO versus OMT, the results of RCTs do not support revascularization over OMT in terms of survival rate or MACE [93,94,95]. The findings from observational studies must be interpreted with caution, as selection bias cannot be excluded; patients with more favorable anatomy, preserved LV function, and fewer comorbidities may be preferentially selected for PCI. Furthermore, operator and institutional expertise play an important role in procedural success, which may not reflect outcomes in broader clinical practice. However, CTO PCI consistently improves angina relief and quality of life, particularly in patients with LAD-CTO or significant ischemia. Future trials with robust designs, adequate sample sizes, and long-term follow-up are essential to clarify which patient subsets derive meaningful prognostic benefit from CTO PCI. The most important prospective randomized trials regarding CTO revascularization are presented in Table 1.

#### 4.1.3. Comparative Studies, Coronary Artery Bypass Grafting Versus Percutaneous Coronary Intervention in Ischemic Heart Failure with Reduced Ejection Fraction

The SYNTAX trial randomized patients with three-vessel disease who qualified for both revascularization strategies (CAGB or PCI) using the Heart Team approach, providing essential insights about long-term outcomes of revascularization. Ten-year survival was reported in the SYNTAX Extended Survival (SYNTAXES) study with a follow-up rate of 93.8%. In the SYNTAX trial, patients previously diagnosed with three-vessel disease or left main CAD were randomly assigned to PCI or CABG. After one year, in the CABG group, the incidence of MACEs was reduced compared to PCI (CABG 12.4% vs. PCI 17.8%; *p* = 0.002), due to increased repeated revascularization in the PCI group (13.5% vs. 5.9%, *p* < 0.001) [44].

After 5 years of follow-up, MACE continued to be significantly increased in the PCI group (37.3% vs. 26.9%; *p* < 0.0001), correlated with a higher rate of repeated revascularization (25.9% vs. 13.7%; *p* < 0.0001) and a higher risk of MI (9.7% vs. 3.8%; *p* < 0.0001). All-cause death was similar (CABG 11.4% vs. PCI 13.9%; *p* = 0.10) as well as stroke (CABG 3.7% vs. PCI 2.4%; *p* = 0.09). There was no significant difference regarding MACEs between PCI and CABG groups (CABG 28.6% vs. PCI 32.1%; *p* = 0.43) in patients with low (≤22) SYNTAX scores, but MACEs were higher in the PCI group for intermediate scores (23–32) (CABG 25.8% vs. PCI 36.0%; *p* = 0.008) and high scores (≥33) (CABG 26.8% vs. PCI 44.0%; *p* < 0.0001) [96]. SYNTAXES was a retrospective study that evaluated long-term survival. All-cause death was similar in the PCI (using first-generation DES) and CABG groups after 10 years of follow-up (PCI 27.46% vs. CABG 23.63%; HR: 1.19; 95% CI: 0.99–1.43; *p* = 0.066). Mortality was increased in the PCI group for patients with three-vessel disease (PCI 28% vs. CABG 21%, HR: 1.42; 95% CI: 1.11–1.81) but not in the left main group (PCI 27% vs. CABG 28%, HR: 0.92; 95% CI: 0.69–1.22; *p* interaction = 0.023). Post hoc analyses in patients with reduced EF who performed PCI or CABG regarding the consequences and the impact of impaired EF on revascularization remain to be established over time. Three-vessel disease was found more frequently in patients with reduced EF compared with mid-range EF and preserved EF (70.2% vs. 63.1% vs. 59.5%; *p* = 0.020) [97].

Patients with reduced EF had worse outcomes compared to those with preserved EF. A significant difference in all-cause mortality for EF groups was first observed at 5 years of follow-up. At 10 years, mortality was 44.0% in patients with reduced EF vs. 31.8% in patients with midrange EF vs. 22.6% in patients with preserved EF (*p* < 0.001). In patients with reduced EF, mortality was higher, especially in the PCI group compared to the CABG group at ten-year follow-up, but without reaching statistical significance (52.9%, versus 39.6%; *p* = 0.054), probably due to the small number of patients enrolled (PCI group: *n* = 77; CABG group: *n* = 91) [97]. Interestingly, the interplay between different EF groups and revascularization strategies observed at 5 years was no longer observed at 10 years [97]. These results suggest that early benefit from CABG in patients with reduced EF becomes less statistically significant over time, particularly in underpowered subgroup analyses.

A critical analysis of the SYNTAX and SYNTAXES reveals several limitations that restrict their applicability to clinical practice. First-generation DESs were used for interventional revascularization during the trial period, limiting the extrapolation of the results to the current period with second and third-generation stents. The subgroup of patients with reduced EF was underpowered, limiting definitive conclusions. Modern medical treatments were not available during the trial period. Furthermore, interventional revascularization techniques for complex PCI, including CTO interventions, have significantly improved since the trial period, potentially influencing outcomes.

From a clinical point of view, it is obvious that the consistent advantage of CABG in patients with three-vessel disease and complex anatomy (SYNTAX score > 23) sustains its role as the preferred revascularization strategy in these patients. For left main CAD, the similar outcomes between CABG and PCI underline the necessity of personalized decision making. In patients with reduced EF, CABG may provide survival benefits, but definitive evidence is lacking due to underpowered analyses.

The Angina with Extremely Serious Operative Mortality Evaluation (AWESOME) trial screened 22,662 patients and enrolled 554 patients with angina refractory to medication, myocardial ischemia, and EF under 35%. Patients enrolled performed percutaneous revascularization or CABG, and the survival rate in the hospital at 30 days was 95% in CABG and 97% in PCI, with no significant difference at 3-year follow-up (79% CABG in the group, 80% in the PCI group) [98]. Despite focusing on high-risk patients, the study had a modest sample size and limited statistical power, restricting definitive conclusions regarding long-term benefit.

The EXCEL trial enrolled 1804 patients who performed PCI (with everolimus drug-eluting stent) or CABG. Among randomized patients, 74 had HF with reduced EF, 152 had HF with mildly reduced EF, and 1578 had HF with preserved EF. Patients with reduced EF experienced a higher rate of complications (death, MI, stroke) compared to patients with HF with mildly reduced and preserved EF at 3 years. The composite primary endpoint showed no differences regarding the type of revascularization, PCI or CABG [99]. The modest number of patients with reduced EF limits definitive conclusions regarding the type of revascularization therapy recommended in these high-risk patients.

According to the FREEDOM (Future Revascularization Evaluation in Patients with Diabetes Mellitus: Optimal Management of Multivessel Disease) clinical trial for diabetic patients with complex CAD, PCI was inferior to CABG with a higher rate of death and MI. Stroke had a higher incidence after CABG, but CABG remains the main strategy for revascularization in this category of patients. Congestive HF NYHA III-IV was an exclusion criterion for this study, so only 3% of the total of 1900 patients had an EF < 40% significantly restricting the applicability of the study to patients with ischemic HF and reduced EF [100].

Observational registry data have provided valuable insights into real-world outcomes. The Swedish Coronary Angiography and Angioplasty Registry (SCAAR) enrolled a large number of patients with ischemic HF, reduced EF, and extensive CAD who underwent coronary angiography. PCI was performed in 56.2% and CABG in 43.8% of cases. After a median follow-up time of 3.9 years, the risk of death was decreased in the CABG group compared to the PCI group (OR 0.62; 95% confidence interval [CI] 0.41–0.96; *p* = 0.031) [101]. While this is an observational study, the discoveries highlight the potential survival benefit of CABG in patients with extensive ischemia and reduced EF.

The same decreased risk in the CABG group was observed after the analysis of the Ontario Registry, where 12,113 patients were enrolled with reduced EF (<35%) and CAD (LAD, left main or MVD) and performed PCI or CABG. The survival benefit was higher in the CABG group, including for the subgroup of patients with one-vessel LAD disease [102].

The APPROACH (Alberta Provincial Project for Outcome Assessment in Coronary Heart Disease) study highlighted the long-term benefits of CABG over PCI in ischemic HF with low EF (HR, 1.21 [95% CI, 1.00–1.46]) [103].

However, these observational registries and retrospective cohort studies have inherent methodological limitations, being subject to selection bias, where treatment allocation (CABG vs. PCI) is influenced by anatomical complexity, comorbidity burden, and operator preference, rather than randomization. Confounding variables may influence treatment effect estimates, making it difficult to establish causality.

A recent meta-analysis, which enrolled >10,000 patients, confirmed the advantage of CABG over PCI in patients with ischemic HF. There was a higher rate of all-cause mortality in the PCI group compared to the CABG group (HR, 1.43 [95% CI, 1.07–1.90]) [104]. Although meta-analyses enhance statistical power, this one was based on observational studies, limiting the cause-and-effect relationship.

All these findings suggest that CABG remains the preferred revascularization strategy in patients with ischemic HF, multivessel CAD, and reduced EF, determined by the long-term survival benefit. However, the applicability of these data to contemporary clinical practice is challenged by limitations inherent in observational studies, outdated PCI techniques in earlier trials, and the underrepresentation of severely reduced EF populations in randomized studies. There is a need for future trials involving patients with ischemic HF and MVD, in the era of contemporary PCI techniques, advanced medical therapy, and with adequately powered subgroups.

### 4.2. The Role of Myocardial Viability Assessment in Guiding Revascularization Strategies

Myocardial viability plays a vital role in the pathophysiology and management of ischemic HF. In patients with CCS, repeated ischemic injury promotes a hibernating and stunned myocardium, leading to fibrosis and adverse LV remodeling [35,36]. This pathophysiologic process underscores the theoretical importance of viability testing to guide revascularization strategies aimed at reversing myocardial dysfunction. Traditionally, the presence and extent of myocardial viability have been considered to be a predictor of LV functional improvement after revascularization.

Variable imaging modalities have been studied in myocardial viability testing. Cardiac magnetic resonance (CMR) and positron emission tomography (PET) are preferred, providing an increased sensitivity and higher spatial resolution. CMR is a key tool in detecting viable heart muscle and myocardial perfusion defects, particularly in territories supplied by a CTO artery [36]. Symptomatic patients with CTO usually have ongoing ischemia despite collateral vessel development. Inducible ischemia and viable myocardium can be depicted by CMR and are useful in establishing the benefit of revascularization therapy [105]. According to Bucciarelli-Ducci et al., all patients who performed CMR and CTO PCI with a viable myocardium had a better quality of life and diminished ischemic burden [106]. A meta-analysis by Allman et al., including 24 small single-center studies involving 573 patients with ischemic LV dysfunction (mean LVEF 33%), demonstrated a strong connection between myocardial viability and improved outcomes. An increased survival rate and enhanced LV function were detected in patients with viable myocardium undergoing revascularization compared to those with medication alone. However, the findings are limited due to the inclusion of non-randomized, retrospective studies with non-standardized treatment protocols and variable methodologies for myocardial viability detection and definition [107].

Although these findings are promising, available RCTs have not consistently confirmed them.

The heart failure revascularization trial (HEART) analyzed 138 patients with HF and reduced EF with a viable myocardium assessed by CMR, who were randomly divided into a conservative treatment group or a revascularization group (PCI or CABG). In the end, only 45 patients underwent revascularization, and, at 59 months of follow-up, a total of 26 patients died. The trial concluded that revascularization in HF with reduced EF and extensive myocardial viability was not superior to the conservative strategy and did not confer a survival advantage [108]. Nevertheless, the small sample size and high crossovers underpowered the study to find significant differences between the treatment arms. Additionally, the findings appear insufficient to influence clinical recommendations, highlighting the need for RCTs in this particularly high-risk population.

In the STICH trial, myocardial viability was assessed by dobutamine stress echocardiography and single photon emission computer tomography (SPECT) in a subgroup of patients but was not a criterion for enrollment. In consequence, there was a heterogeneity in viability status among randomized patients that reduced the ability to discern which subgroups had the greatest benefit from CABG. Despite the fact that, in the long term, the presence of a viable myocardium correlated with a marginal improvement of LVEF, this was not associated with treatment allocation and did not impact the overall survival. The viability STICH sub-study had many limitations: the inhomogeneity of imaging protocols for viability assessment, the variable definition of viability thresholds, and the absence of more accurate imaging tools as CMR or PET, for viability evaluation [49].

In the REVIVED-BCIS2 trial, viability was assessed predominantly via CMR before PCI. The amount of viable myocardium did not correlate with the outcome of revascularization. The majority of enrolled patients presented limited viable myocardium, and crossovers between treatment arms further complicated interpretation [18].

In the F-18-Fluorodeoxyglucose Positron Emission Tomography Imaging-Assisted Management of Patients With Severe Left Ventricular Dysfunction and Suspected Coronary Disease (PARR-2) trial, 430 patients with diminished EF and ischemic CAD were randomly assigned to the treatment guided by imaging (positron emission tomography [PET] with fluorodeoxyglucose) or standard therapy [109]. After a 5-year follow-up, PET-assisted management did not demonstrate a significantly lower rate of cardiac events compared to a standard protocol in patients with reduced EF and CAD. However, a subgroup analysis of patients who adhered to PET guidance, particularly those with extensive viability, revealed substantial benefits in terms of the primary outcome. A large proportion of patients in both groups underwent surgical revascularization, and the results indicated that those with extensive myocardial viability derived the greatest benefit. However, treatment crossover and variable adherence undermined the study’s ability to conclusively validate viability-guided decision making [110].

The EXPLORE-CTO inclusion criteria did not demand viability testing, potentially leading to the inclusion of patients unlikely to benefit from revascularization due to limited viable myocardium. The sample size was modest, 304 patients, and CMR was performed in 180 patients, narrowing generalizability. In the subgroup with LAD-CTO and viable myocardium, an improvement in LVEF was observed, suggesting that viability and LAD involvement may influence outcomes [75,76,77,78,79].

A recent meta-analysis including all the evidence extracted from the major RCTs performed in patients with ischemic cardiomyopathy found that the viability-guided revascularization strategy had no significant impact on survival compared to a standard-of-care strategy [111].

In summary, the current evidence regarding the clinical utility of myocardial viability testing in guiding revascularization for ischemic HF is controversial. While observational studies and meta-analyses suggest survival benefits and improved LV function in patients with viable myocardium, RCTs have not consistently confirmed these findings. Variability in imaging modalities (PET, CMR, SPECT), in defining viability, and in the extent of ischemia complicates the interpretation and external validity of the data. Moreover, viability alone may not sufficiently predict clinical outcomes without integrating factors such as ischemia burden, scar extent, and patient comorbidities. Future large-scale, well-powered RCTs using standardized modern imaging techniques and stratified patient selection are needed to clarify the clinical utility of viability-guided revascularization strategies, particularly in patients with MVD and complex coronary anatomy.

### 4.3. Survival Outcome with Revascularization Compared to Optimal Medical Therapy

Is revascularization improving survival outcomes in patients with HF compared to OMT? The answer is a subject of ongoing debate, with RCTs providing mixed results and observational studies showing potential benefits in selected populations.

The RCT—the international study of comparative health effectiveness with medical and invasive approaches—Ischemia Trial failed to demonstrate the efficacy of routine revascularization in patients with stable angina compared to medical therapy, with adverse events like cardiovascular death and MI similar regardless of the degree of ischemia or complexity of CAD [112]. In this study of 5179 randomized patients, 398 had HF and LV dysfunction (177 patients with LVEF > 45%, 221 patients with LVEF 35–45%). This particular subgroup had more comorbidities compared with the group without HF, and the incidence of cardiac death, repeated hospitalization for HF, unstable angina, and non-fatal MI was higher. Invasive strategy was associated with a reduced primary outcome compared to conservative therapy (17.2% vs. 29.3%) [112]. Although the sample size was small and long follow-up data are missing, these findings suggest that there are potential benefits for interventional revascularization in selected HF patients.

The Revascularization for Ischemic Ventricular Dysfunction (REVIVED-BCIS2), a prospective multicentric randomized open-label trial that enrolled 700 patients, compared the benefits of PCI added to OMT versus OMT alone in patients with severe ischemic LV dysfunction, an EF of 35% or less, and extensive CAD suitable for PCI. For 3,4 years, the primary endpoint (composite of death from any cause or hospitalization) was similar in all groups (37.2% versus 38%, HR 0.99, 95% CI 0.78–1.27; *p* = 0.96) [18]. Secondary outcomes were focused on EF and demonstrated no differences between the groups at the 6- and 12-month follow-ups. Quality of life improved in the PCI group at 6 and 12 months, although the difference diminished by 24 months. The extent of dysfunctional yet viable myocardia was not associated with revascularization outcomes [18]. Similarly, no correlation was found between inducible ischemia and study treatment. All-cause death and hospitalization for HF were similar after 41 months in patients treated with OMT alone compared to patients in whom OMT was combined with PCI to restore myocardial perfusion in areas with preserved viability, including in the group of patients with significant left main or proximal LAD stenosis [112]. Although the trial enrolled patients with reduced EF (mean LVEF~27%), a significant proportion had a limited viable myocardium, as patient selection was based on investigator discretion using CMR or dobutamine stress echocardiography, with a minimum viability threshold of four segments. Patients with large ischemia territories or important myocardial scars were underrepresented, limiting the applicability of the findings to the entire HF population. High rates of crossovers were allowed in this trial; 19.9% of patients in the OMT group underwent PCI. Considerable crossovers underestimate and influence the detection of differences in clinical endpoints, confounding the results and making it difficult to assess the real benefit of PCI in the target population. The trial lacked sufficient statistical power to demonstrate a definitive advantage of revascularization regarding all-cause mortality and HF hospitalizations. On top of that, the event rates were less than anticipated, restricting the ability to detect substantial differences between the groups. The short median follow-up of 3,4 years can be insufficient to detect late survival benefits in a target population with CCS. Even if the trial did not show a benefit of interventional revascularization regarding mortality or HF hospitalization, the role of PCI should not be completely denied in all HF patients. The findings of the study are primarily applicable to patients resembling those enrolled in the study and may not be extrapolated to individuals with extensive ischemia who could have greater benefit from revascularization.

Deeper analyses have revealed that PCI was associated with modest but statistically significant early improvements in angina relief and quality of life scores, but this benefit diminished over time [113]. Although the trial did not reveal LVEF improvement in the PCI group, some subgroup trends showed that patients with less extensive myocardial scar or higher degrees of viability may experience more symptomatic benefit. The results regarding hard outcomes were neutral, and this raised important questions about whether myocardial viability alone is sufficient to predict functional recovery.

The REVIVED findings highlight that, in the era of contemporary guideline-directed medical therapy, the incremental benefit of PCI in stable ischemic HF with reduced EF may be limited to selected patient subsets, sustaining a personalized, symptom-based approach combined with imaging and multidisciplinary evaluation.

The Revascularization and Outcomes in Ischemic Left Ventricular Dysfunction After Heart Failure Admission (RevascHeart) study, a recent cohort study conducted from 2012 to 2023, enrolled 408 patients with HF and EF of 40% or less with documented ischemia. Patients were divided into two major groups: revascularized patients (CABG or PCI) and the guideline-directed medical therapy group. The mortality was higher in the revascularized group (100 patients compared to 44). In both groups, there was a significant positive remodeling of the LV with improvement of EF, slightly superior in the revascularized group. Revascularization was not superior to OMT in patients with HF and LV dysfunction, but further studies are needed to establish the potential role of revascularization in ischemic patients with HF [114].

The APPROACH registry included patients with HF (most of them with EF less than 50%) who performed coronarography. From a number of 4228 patients who were eligible for the registry, 2538 were revascularized (52.5% with CABG and 48.5% with PCI). The mortality rate was higher in the group without revascularization therapy (21.6%) compared to the revascularization group (11.8%). CABG had a better survival benefit than PCI, but, overall, the conclusion was that patients with ischemic HF who underwent revascularization had a better outcome and an increased survival rate. When interpreting the results, we must consider that the data from this registry are from the prior OMT era, which can limit its applicability to current practices [115].

A small retrospective study conducted on 76 patients with severe ischemic HF who underwent interventional revascularization showed, at 6–180 months of follow-up, an increase in LVEF, a decrease in LV volumes and of pulmonary hypertension, an improved survival, and a delayed heart transplantation in 74% of the patients [116]. While discoveries are promising, the small sample size and retrospective design restrict the power of causal inference. Furthermore, follow-up intervals are not constant, making it harder to generalize outcomes or give credit only to the intervention.

The results of the ongoing trial regarding the role of revascularization in ischemic HF are not yet released. RESTORE-PCI (Revascularization versus Medical Treatment in Patients with Ischemic Left Ventricular Dysfunction) is an ongoing trial aiming to compare the effect of PCI with OMT in patients with ischemia and LV dysfunction. At the time of writing, there are 900 patients enrolled with HF, NYHA class III, and EF under 40% and at least 50% stenoses on the coronary arteries. The first hypothesis is that PCI plus OMT reduced the risk of MACEs and hospitalization for HF more effectively than drug therapy alone in ischemic cardiomyopathy [117]. This study targets the populations underrepresented in previous studies, promising clear evidence regarding the role of interventional revascularization in the context of modern HF therapy.

Despite advances in research, the survival benefit of revascularization compared to OMT in ischemic HF remains unclear. Based on the results of the available RCTs and guidelines recommendations, CABG remains the first choice of revascularization in ischemic HF. But the evidence regarding the role of CABG dates mainly from the period before the consistent advances in the treatment of HF. The gap between CABG and OMT has probably changed, and we need new RCTs that should compare PCI and CABG versus OMT in the era of modern HF management.

## 5. Coronary Microvascular Dysfunction in Ischemic Heart Failure

Coronary microvascular dysfunction (CMD) is increasingly recognized as a significant contributor to myocardial ischemia, particularly in patients with HF with preserved EF, but also plays a role in selected cases of HF with reduced EF. CMD involves structural and functional abnormalities in the coronary microcirculation, including endothelial dysfunction, vascular smooth muscle cell impairment, microvascular rarefaction, and capillary remodeling, leading to reduced coronary flow reserve and myocardial ischemia, even in the absence of obstructive epicardial stenoses. Functional CMD mechanisms include impaired vasodilation due to reduced bioavailability of nitric oxide (NO), prostaglandins, and endothelium-derived hyperpolarizing factors, along with increased release of vasoconstrictor mediators such as endothelin-1. Structural alterations, including smooth muscle hypertrophy, perivascular fibrosis, and capillary rarefaction, further reduce myocardial perfusion and contribute to diastolic dysfunction, interstitial fibrosis, and adverse LV remodeling. It is well known that comorbidities associated with systemic and coronary inflammation, such as diabetes or chronic kidney disease, hypertension, and obesity, have an important role in promoting endothelial dysfunction and CMD, leading to HF, particularly HF with preserved EF.

CMD represents an ischemic phenotype where conventional revascularization strategies may have limited therapeutic benefit, as these procedures do not address microvascular dysfunction. This explains the limited symptomatic and functional recovery in certain patients despite a successful epicardial revascularization [118,119,120].

Diagnostic methods of CMD include non-invasive coronary flow measurements, such as PET, CMR perfusion imaging, and transthoracic Doppler LAD echocardiography, and invasive methods such as the guidewire-based coronary flow reserve and/or microcirculatory resistance measurements. In the current ESC guidelines, invasive testing of CMD has a class II-a recommendation, while non-invasive testing holds a class II-b recommendation in CCS [1]. Incorporating CMD assessment can prevent unnecessary revascularization in patients where CMD, rather than epicardial stenosis, is the principal driver of ischemia.

Currently, no guideline-directed therapies specifically target CMD. However, several pharmacologic agents that improve endothelial function, modulate vascular inflammation, and optimize metabolic demand–supply balance have shown promise in CMD. SGLT2Is and ARNIs, important pillars in the treatment of HF with reduced EF, may also have microvascular protective effects. SGLTIs can inhibit pro-inflammatory cytokines and ROS production and improve endothelial function [121]. ARNIs are further studied regarding the potential effect in CMD using CMR in patients with HF and preserved EF in the Study of Sacubitril/ValsarTan on MyocardIal OxygenatioN and Fibrosis in Heart Failure with Preserved Ejection Fraction (PRISTINE-HF) [122]. Statins have anti-inflammatory effects and ameliorate endothelial dysfunction, improving endothelium-dependent coronary relaxation [119]. Third-generation betablockers (nebivolol, carvedilol) are essential therapeutic agents in HF with reduced EF, enhancing endothelial function, reducing myocardial oxygen consumption, and increasing diastolic perfusion time [123], leading to improved exercise performance. ACEIs, in particular, Quinapril, were demonstrated in the Trial on Reversing Endothelial Dysfunction (TREND) study to have a beneficial impact on endothelial function [124]. These beneficial effects of ACEIs on CMD were considered to derive from their ability to inhibit coronary vasoconstriction and superoxide production while enhancing the release of NO from the endothelium. Glucagon-Like Peptide Receptor Agonists (GLP-1 RAs) decrease reactive oxygen species (ROS) production and oxidative stress and modulate inflammation, potentially improving CMD [125]. Lifestyle interventions targeting CMD drivers, such as exercise, weight control, and smoking cessation, remain essential elements [122].

Future clinical trials must focus on CMD-stratified patient cohorts to elucidate which subgroups may benefit from medical therapy alone versus revascularization. A combination strategy addressing both epicardial and microvascular disease may represent a promising therapeutic avenue, moving toward precision medicine in ischemic HF management.

## 6. Contemporary Optimal Medical Therapy in Ischemic Heart Failure

According to ESC guidelines of heart failure 2021 and the updated version in 2023, there are four pillars of treatment for HF with reduced EF: ACEIs or ARNIs, betablockers (BBs), mineralocorticoid receptor antagonists (MRAs), and SGLT2Is [37,126].

ACEIs remain the first-line treatment for chronic HF, provided there are no contraindications. Their effectiveness is particularly well-established in ischemic HF, where they have been shown to significantly reduce both mortality and morbidity [127,128,129,130,131,132]. The Heart Outcomes Prevention Evaluation (HOPE) study enrolled 9245 patients with high blood pressure, HF, and microalbuminuria randomized to 10 mg of ramipril daily or placebo for 4.5 years. In the ramipril group, the incidence of HF events was significantly reduced [133]. The Survival of Myocardial Infarction Long-Term Evaluation (SMILE) study highlighted that the administration of zofenopril in patients with anterior MI significantly reduced the incidence of severe congestive HF and was associated with an improved survival rate [134]. In cases of intolerance (cough, history of angioedema) to ACEIs, angiotensin receptor blockers (ARBS) can be used as an alternative therapy [37,135]. Both ESC and American Heart Association (AHA) guidelines included ACEIs as a class of recommendation I, level of evidence A in the management of HF with reduced EF [13,37].

ARNIs proved to be superior to ACEIs in terms of hospitalizations and death in patients with reduced EF in the Prospective Comparison of ARNI with ACEI to Determine Impact on Global Mortality and Morbidity in Heart Failure-PARADIGM-HF, a prospective RCT [37,136,137,138,139,140]. Consequently, both the AHA and ESC guidelines recommend ARNIs as a replacement therapy for ACEIs or ARBs in HF patients with reduced EF who remain symptomatic despite initial therapy. Furthermore, ARNIs may be considered as first-line therapy instead of ACEIs, provided there are no contraindications [126,136]. Comparison of Sacubitril-Valsartan versus Enalapril on Effect on NT-proBNP in patients Stabilized from an Acute Heart Failure Episode PIONEER-HF trial showed that, in patients with HF with reduced EF hospitalized for an acute decompensation, ARNIs improved health status, LV remodeling, and decreased N-terminal pro B-type natriuretic peptide (NT-proBNP) levels compared to enalapril [141].

Initiation of sacubitril/valsartan in hemodynamically stabilized patients in hospital or early after discharge; the TRANSITION trial enrolled patients with worsening HF and demonstrated that early initiation of ARNI therapy is safe and well tolerated in the absence of contraindications [142].

Along with ACEIs/ARNIs, BBs (especially bisoprolol, sustained-release metoprolol succinate, and carvedilol) showed a lower mortality and improvement of EF and symptoms in hemodynamically stabilized patients [143,144,145]. The Carvedilol or Metoprolol European Trial (COMET trial) made a comparison between carvedilol and metoprolol regarding mortality rates in patients with chronic HF, NYHA class II-IV, and carvedilol came forward with a better outcome without a major difference in side effects [146]. The Cardiac Insufficiency Bisoprolol II-CIBIS-II, a multicenter double-blind randomized trial, showed the effects of bisoprolol in patients with HF NYHA III-IV with an EF of 35% or less and demonstrated an increase in survival rate [147]. Metoprolol controlled release/extended release in patients with severe heart failure—MERIT-HF—highlights the efficiency of metoprolol, showing reduced hospitalization, improved quality of life, and NYHA functional class [148]. AHA and ESC guidelines recommend BBs in all patients with stable HF and reduced EF unless there are contraindications (Class I Level A) [37,135].

Adding MRAs in ischemic HF therapy determined a lower rate of mortality and hospitalization in HF patients, unless there are contraindications: impaired renal function or hyperkalemia [126,149,150,151,152,153,154]. Trials such as the Randomized Aldactone Evaluation Study—RALES, Eplerenone in Patients with Systolic Heart Failure and Mild Symptoms (EMPHASIS-HF), and Eplerenone post-AMI Heart Failure Efficacy and Survival Study (EPHESUS) showed that MRAs improve symptomatology and decrease mortality in patients with HF and reduced EF [155,156,157]. AHA and ESC guidelines include MRAs as a class of recommendation I level of evidence A in HF with reduced EF [135,158].

SGLT2Is are the newest therapy in HF, in addition to the medication already mentioned [159,160,161,162]. Dapaglifozin in patients with Heart Failure and Reduced Ejection Fraction (DAPA-HF) trial and Cardiovascular and Renal Outcomes with Empaglifozin in Heart Failure (EMPEROR-REDUCED) demonstrated that SGLT2Is decrease cardiovascular death and improve prognosis in patients with HF with reduced EF and NYHA class II-IV [163,164]. AHA and ESC guidelines include SGLT2Is as a class of recommendation I level of evidence A [127,135].

**Figure 3:**
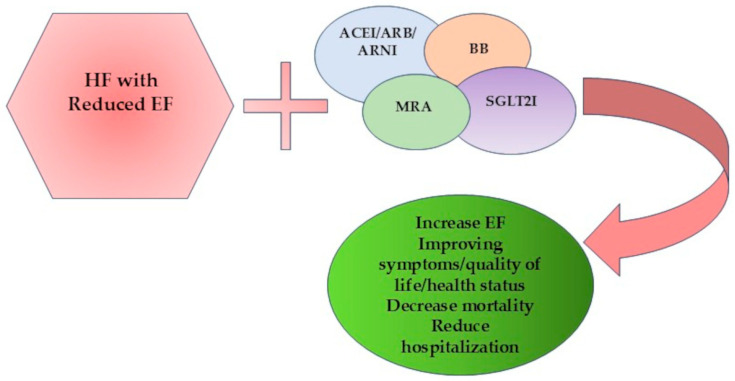
Benefits of optimal medical therapy in heart failure. Legend: HF—heart failure; EF—ejection fraction; ACEI—angiotensin-converting enzyme inhibitor; ARB—angiotensin receptor blocker; ARNI—angiotensin receptor-neprilysin inhibitor; MRA—mineralocorticoid receptor antagonist; SGLT2I—sodium glucose co-transporter 2 inhibitor; and BB—betablocker.

Optimal medical therapy of HF with reduced EF evolved over the years, with recent trials providing strong evidence that led to major changes in the management of this form of HF. Collectively, these studies showed a benefit in outcome by reducing mortality, morbidity, and hospitalization rates. Gaps in evidence continue to exist, especially in HF with mildly reduced EF, HF with preserved EF, advanced age, frailty, and renal dysfunction, where further studies are required. OMT should no longer be viewed as a passive comparator but as an active, evolving standard of care that redefines the clinical relevance and the supplementary benefit of revascularization strategies in contemporary clinical practice. Figure 3 summarizes the effects of optimal medical therapy in HF with reduced EF.

The following table summarizes current evidence derived from available RCTs regarding main treatment modalities in HF with reduced EF, focusing on key outcomes (Table 2).

## 7. Guidelines and Recommendations

Before the introduction of modern OMT, the RCTs and meta-analysis results reported a survival improvement for CABG in patients with MVD and left main disease, especially in patients with low EF [1,165,166,167]. In consequence, CABG is considered superior to medical therapy and PCI in patients with MVD and ischemic HF, particularly in those with diabetes and high-complexity CAD [1,168,169].

In 2018 ESC guidelines for myocardial revascularization, coronary revascularization (PCI or CABG) was considered the first choice in patients with ischemic HF because of increased benefit in survival over medical therapy, so there is a class of recommendation (COR) I level of evidence (LOE) B for patients with severely reduced EF (<35%) and CAD appropriate for revascularization [17]. The revascularization strategy, CABG or PCI, is decided by the Heart Team according to the clinical condition, severity, and complexity of CAD, the probability to achieve CR, the presence of viable myocardium, and significant valvular disease and comorbidities [17]. For patients with MVD and acceptable surgical risk, CABG is recommended COR I LOE B, preferred especially in younger patients with extensive CAD and in diabetic patients. PCI is an alternative when CR can be achieved, especially for older patients without diabetes (COR II-a LOE C) [17].

In the 2024 ESC CCS guidelines, it is recommended that the decision to perform myocardial revascularization versus medical therapy alone in patients with CCS and reduced LVEF (<35%) should be based on the Heart Team decision after a meticulous assessment of the severity and extent of coronary arteries lesions, of the correlation between CAD and LV dysfunction, and of the comorbidities and life expectancy (COR I LOE-C) [1]. In ischemic HF with reduced EF, every new ischemic event worsens LVEF and can diminish survival [170]. For prognostic improvement in patients with acceptable surgical risk, CABG remains preferred over medical therapy in MVD and reduced LVEF, with a recommendation COR I LOE B [1]. In patients with high surgical risk or not operable, PCI may be considered as an alternative (COR II-b LOE B) [1]. For reducing the risk of complications and to increase the success of CR during a high-risk PCI for complex CAD in patients with HF and reduced EF, mechanical cardiac support, a preferred microaxial flow pump, is recommended [1,171,172]. In symptomatic patients, despite OMT, with persisting angina or angina equivalents, myocardial revascularization is recommended to improve symptoms (COR I LOE A) [1]. The most appropriate therapy will be defined within a shared decision between patients and healthcare professionals after a detailed discussion about the disease and the methods of treatment and after considering patient preferences (COR I LOE C) [1].

The 2021 ACC/AHA/Society of Cardiovascular Angiography and Interventions (SCAI) guidelines for coronary artery revascularization recommend CABG beyond conservative therapy in patients with MVD and impaired LV systolic function (EF < 35%) as COR 1 LOE B-R for improved survival. Regarding stable ischemic cardiomyopathy with MVD and EF between 35 and 50%, the indication for CABG (including a left mammary internal artery on LAD) is reasonable to improve survival with COR 2a LOE B-NR recommendation [173]. Although there are no RCTs that proved a survival advantage of PCI over OMT in patients with stable chronic ischemic disease, PCI may have a benefit over medical therapy in patients with an indication for CABG but a prohibitive surgical risk [173].

The 2022 AHA/ACC/Heart Failure Society of America (HFSA) guidelines for the management of HF recommend CABG and OMT in patients with ischemic cardiomyopathy and EF equal to or less than 35% as COR 1 LOE B-R, with benefits on symptoms, hospitalizations, and long-term all-cause mortality [138].

**Figure 4:**
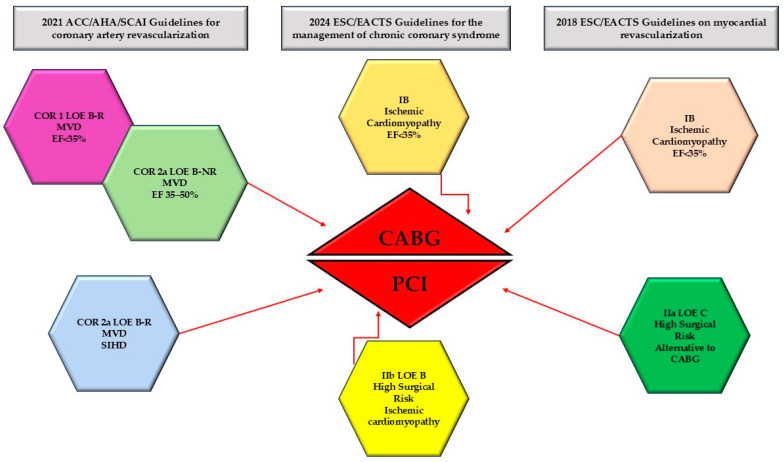
ACC/AHA/SCAI/ESC/EACTS guidelines recommendations for CABG and PCI. Legend: ACC—American College of Cardiology; AHA—American Heart Association; SCAI—Society for Cardiovascular Angiography & Interventions; ESC—European Society of Cardiology; EACTS—European Association for Cardiothoracic Surgery; CABG—coronary artery bypass grafting; EF—ejection fraction; MVD—multivessel disease; PCI—percutaneous coronary intervention; and SIHD—stable ischemic heart disease.

The recommendations for revascularization from current guidelines are depicted in Figure 4 [1,17,173].

**Figure 5:**
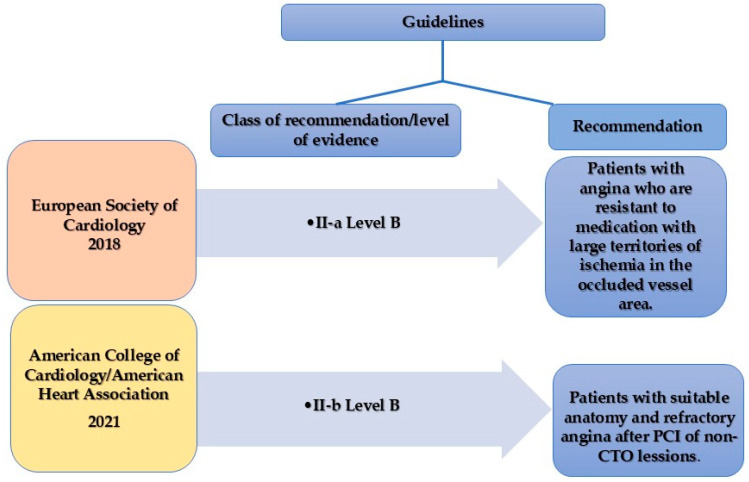
Guideline recommendations for PCI-CTO. Legend: PCI—percutaneous coronary intervention; CTO—chronic total occlusion.

Regarding CTO, the 2018 ESC guidelines on myocardial revascularization recommend CTO revascularization as a COR II-a, LOE B in symptomatic patients refractory to medical therapy [17]. The AHA guidelines from 2017 recommended CTO-PCI only in patients with angina when revascularization can be performed by an experienced team [174]. However, the recent American guidelines released in 2021 consider the benefit of CTO-PCI uncertain even in patients with suitable anatomy or angina, so they downgraded the recommendation from COR 2a to 2b LOE B-R [173]. Figure 5 illustrates recommendations from current guidelines regarding CTO.

## 8. Future Perspectives

Currently, CABG remains the best choice for complete myocardial revascularization in patients with ischemic HF, being supported by guidelines [138] and with evidence of improved survival. However, future RCTs are needed to establish whether CABG in addition to OMT has a more favorable impact on outcome vs. OMT alone in the era of modern medical treatment.

Hybrid coronary revascularization (HCR) is a novel strategy that combines a minimally invasive CABG and PCI to improve outcomes in selected patients with complex CAD. HCR implies a minimally invasive surgical graft of the left internal mammary artery to the LAD artery, followed by PCI for non-LAD lesions. This result will be a combination of the long-term patency benefits of surgical grafting with minimal procedural invasiveness and surgical trauma, which is especially important in patients with reduced LV function [175,176].

In ischemic HF with reduced EF, HCR may offer an alternative to conventional CABG or multivessel PCI, particularly for patients at high risk for full sternotomy or prolonged cardiopulmonary bypass due to comorbidities, frailty, or poor ventricular function. Observational studies and retrospective analyses reveal that HCR is feasible and may lead to favorable outcomes in terms of symptom control, procedural safety, and LV function preservation while providing reduced hospitalization and faster recovery times compared to CABG [177].

However, RCTs evaluating HCR in patients with HF and reduced EF are not available. Furthermore, HCR presents multiple logistical and technical challenges and requires institutional expertise in minimally invasive cardiac surgery and coordination between surgical and interventional teams with timing of staged procedures (simultaneous versus delayed PCI).

HCR represents a promising direction in the personalization of coronary revascularization in ischemic cardiomyopathy. For selected patients, with suitable coronary anatomy and isolated LAD disease amenable to surgical grafting, HCR may offer a balanced approach that avoids the extremes of either full surgical or full PCI [178]. Further prospective studies are necessary to evaluate its role in these high-risk patients and to determine its impact on long-term survival and functional recovery.

Interventional cardiology evolves daily both technically (FFR, intravascular imaging) and in terms of materials (new stents) used, but the question regarding the survival benefit over guideline-directed medical therapy nowadays remains unanswered [179,180]. Further studies are mandatory to offer the best management approach comparing CABG vs. PCI in the new era of modern medicine for HF with reduced EF.

Many gaps in evidence should be addressed in future research directions. The role of myocardial viability testing remains controversial; additional studies are required to demonstrate whether the assessment of viable myocardium will have a significant impact on EF, positive cardiac remodeling, and patient outcome [181]. Furthermore, the evaluation of myocardial viability and ischemia burden should be based on a standardized protocol followed by imaging-guided therapy to assess the impact of viability testing on clinical outcome. Future studies should also focus on the classification of patients based on comorbidity burden (e.g., frailty, diabetes, CKD) and the evaluation of the impact of CR (functional and anatomical) on clinical outcomes. Long-term follow-up studies are essential to more accurately assess survival benefits, particularly for PCI strategies. Furthermore, future research should also focus on the development of personalized therapy, specifically a combination therapy approach, integrating pharmacologic OMT with tailored revascularization strategies, accounting for patient anatomy, functional status, and preferences.

In patients with ischemic HF with preserved or mildly reduced EF, future studies should evaluate whether revascularization brings an additional benefit over medical therapy [182].

Identifying the category of patients who will benefit the most after revascularization requires further research that should include patients with ischemic HF and impaired EF, tested for myocardial viability, and divided into vascularized and non-vascularized groups for a more accurate assessment of the differences regarding survival and outcomes.

## 9. Conclusions and Clinical Relevance

Despite the remarkable advances in OMT followed by an important improvement in outcome, ischemic HF remains associated with a worse prognosis and high risk of future major cardiac events. The role of myocardial revascularization in the era of modern HF pharmacotherapy remains controversial.

Clinical take-home points for multidisciplinary care teams:

1. CABG remains the preferred revascularization strategy in patients with MVD, suitable coronary anatomy, and acceptable surgical risk, especially in diabetics, younger patients, and those with concomitant significant mitral regurgitation. These recommendations are derived from trials conducted before the widespread use of contemporary pharmacological HF therapies.
2. PCI has not demonstrated a survival benefit over OMT in randomized trials for ischemic HF. However, PCI may be a reasonable revascularization strategy in patients with prohibitive surgical risk, patients with persistent angina despite OMT, particularly when angina predominates over HF-related symptoms, and cases with documented myocardial viability in territories amenable to PCI.
3. The role of CTO PCI is not established; the current guidelines recommend it for symptomatic improvement in patients with refractory angina or extensive ischemia (LAD territory) when anatomical factors are favorable.
4. Viability testing, though not mandatory, can aid in the revascularization decision-making process, especially in selecting borderline surgical candidates and in high-surgical-risk patients. When HF symptoms predominate, evaluation of myocardial scar burden versus viable hibernating myocardium will help in deciding between OMT and a revascularization strategy.
5. OMT remains the essential therapeutic element of HF management, with ARNI, BB, MRA, and SGLT2I forming the main pillars, with clear evidence of improving survival and reducing hospitalizations.
6. The optimal revascularization strategy in ischemic HF must be individualized. Elements as anatomical complexity, myocardial viability, comorbidities, and patient-centered factors should be assessed within a multidisciplinary Heart Team framework. The current evidence is insufficient to definitively determine the role of PCI in ischemic cardiomyopathy; future research should fill the gap in knowledge with rigorously designed, contemporary trials.

**Figure 6:**
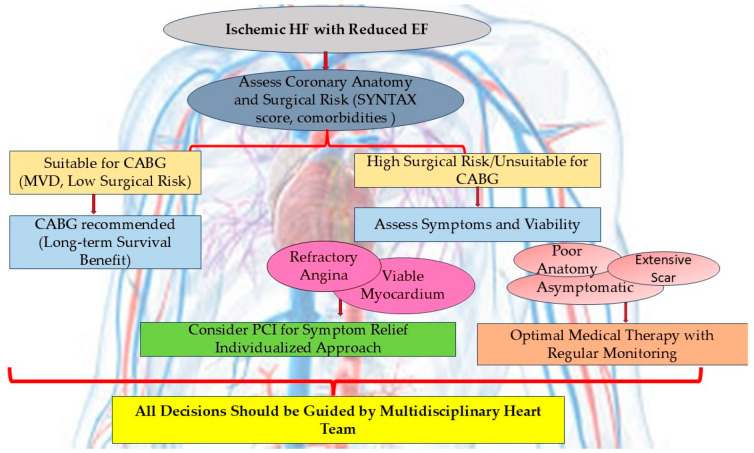
Algorithm for revascularization decision making in ischemic HF with reduced EF. Legend: HF—heart failure; EF—ejection fraction; CABG—coronary artery bypass grafting; MVD—multivessel disease; and PCI—percutaneous coronary intervention.

To translate these conclusions into clinical practice, we propose a revascularization decision-making algorithm that stratifies patients based on anatomical, functional, and clinical parameters, facilitating a structured and evidence-based approach for selecting the most appropriate revascularization strategy in ischemic HF (Figure 6).

## Figures and Tables

**Table 1:** Randomized prospective trials regarding CTO revascularization.

Study	Time	Inclusion Criteria	Exclusion Criteria	No. Pts.	Symptoms/ EF	Mortality/ Outcome/ MACE	Ref.
DECISION-CTO	2010–2016	Silent ischemia Stable angina ACS CTO > 2.5 mm	EF < 30% CTO 3 different vessels Severe comorbidities	834	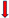 Angina 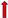 Quality of life 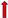 EF	No difference	[83]
EURO-CTO	2012–2015	CTO > 2.5 mm CTO > 3 months	Intolerance to dual antiplatelet therapy Need for elective non-cardiac surgery	396	NYHA class improved at 12 months in PCI groups	No difference	[32]
EXPLORE-CTO	2007–2015	CTO > 2.5 mm CTO in non-infarcted related artery after PCI for STEMI	Hemodynamic instability Valvular disease requiring surgery AF Severe renal insufficiency	304	CTO-LAD 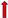 EF at 4 months follow-up 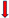 Angina	No difference	[31]
IMPACTOR-CTO	2010–2014	RCA-CTO	Unsuccessful PCI-CTO	94	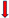 Angina Improved symptoms	No difference	[33]

Legend: EF—ejection fraction; CTO—chronic total obstruction; PCI—percutaneous coronary intervention; ACS—acute coronary syndrome; STEMI—ST-elevation myocardial infarction; RCA—right coronary artery; AF—atrial fibrillation; LAD—left anterior descending artery; MACE—major adverse cardiac event; OMT—optimal medical therapy; 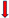
—decrease and 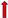
—increase.

**Table 2:** Comparative outcomes of PCI, CABG, and OMT in ischemic HF with reduced EF.

Treatment	Mortality	EF Improvement	MACE	Symptoms Relief And QOL	Hospitalization Rate
PCI	No consistent benefit over OMT	Limited/no significant improvement over OMT	No benefits over OMT	QOL improved at 6–12 months/no difference at 24 months	No significant reduction over OMT
CABG	Consistent long-term benefit over OMT Long-term benefit over PCI in MVD/SYNTAX score > 23	Improvement in selected groups with severe LV dysfunction	Reduced in MVD/SYNTAX score > 23	Improved	Reduced in the long term
Modern OMT	Consistent benefit	Improvement	Reduced	Improved/limited in refractory angina	Reduced

Legend: CABG—coronary artery bypass grafting; MVD—multivessel disease; OMT—optimal medical therapy; PCI—percutaneous coronary intervention; LV—left ventricular; and QOL—quality of life.

## Supplementary Materials

The following supporting information can be downloaded at https://www.mdpi.com/article/10.3390/medicina61081451/s1, Figure S1: PRISMA flow diagram.

## Abbreviations

The following abbreviations are used in this manuscript:

ACS	Acute coronary syndrome
ACC	American College of Cardiology
ACEIs	Angiotensin-converting enzyme inhibitors
AHA	American Heart Association
ARB	Angiotensin receptor blocker
ARNIs	Angiotensin receptor-neprilysin inhibitors
BB	Betablocker
CAD	Coronary artery disease
CABG	Coronary artery bypass grafting
CCS	Chronic coronary syndrome
CMD	Coronary microvascular dysfunction
CMR	Cardiac magnetic resonance
COR	Class of recommendation
CR	Complete revascularization
CTO	Chronic total occlusion
CXA	Circumflex artery
EF	Ejection fraction
ESC	European Society of Cardiology
EACTS	European Association for Cardiothoracic Surgery
FFR	Fractional flow reserve
GLS	Global longitudinal strain
GLP-1 Ras	Glucagon-like peptide receptor agonists
HCR	Hybrid coronary revascularization
HF	Heart failure
HFSA	Heart Failure Society of America
ICR	Incomplete revascularization
IVUS	Intravascular ultrasound
J-CTO	Japanese chronic total occlusion
LAD	Left arterial descending
LOE	Level of evidence
LV	Left ventricle
MACE	Major adverse cardiovascular event
MeSH	Medical Subject Heading
MI	Myocardial infarction
MVD	Multivessel disease
MR	Mitral regurgitation
MRA	Mineralocorticoid receptors
NO	Nitric oxide
NT-proBNP	N terminal proB-type natriuretic peptide
Non-IRA	Non-infarct-related artery
NSTEMI	Non-ST-elevation myocardial infarction
NYHA	New York Heart Association
OMT	Optimal medical therapy
PCI	Percutaneous coronary intervention
PET	Positron emission tomography
RCA	Right coronary artery
RCTs	Randomized controlled trials
ROS	Reactive oxygen species
SCAI	Society for Cardiovascular Angiography & Interventions
SGLT2Is	Sodium glucose co-transporter 2 inhibitors
STEMI	ST elevated myocardial infarction
