# Supplementary figures and images for: The Role of Myocardial Revascularization in Ischemic Heart Failure in the Era of Modern Optimal Medical Therapy
Source: Medicina (Kaunas). 2025 Aug 12;61(8):1451. doi: 10.3390/medicina61081451 (PMC12388460; doi:10.3390/medicina61081451)

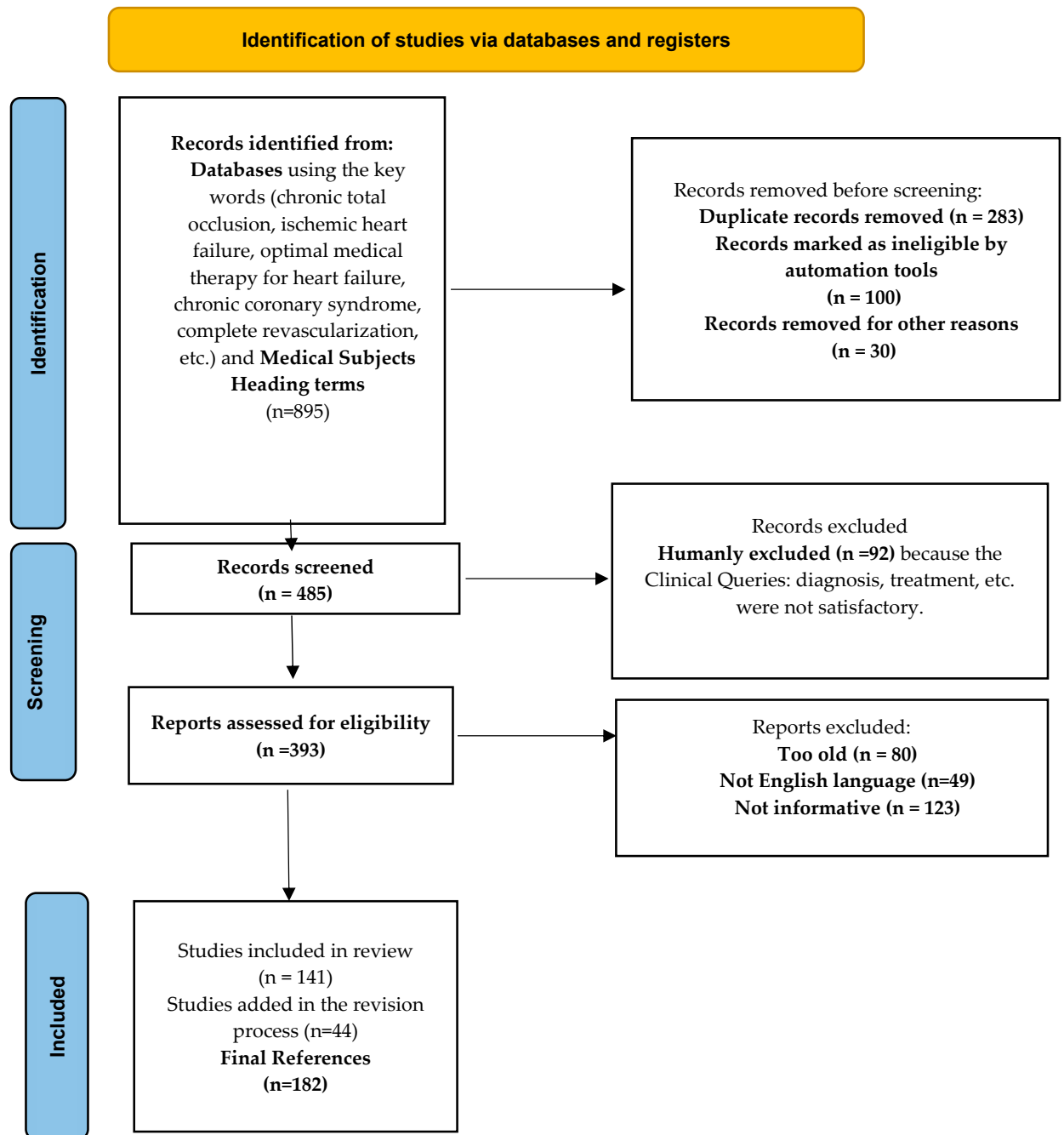

Figure S1: PRISMA flow diagram

Supplement: Supplementary file 1 [file medicina-61-01451-s001.zip › medicina-3797568-supplementary.pdf]
